# Postoperative Tinnitus After Vestibular Schwannoma Surgery Depends on Preoperative Tinnitus and Both Pre- and Postoperative Hearing Function

**DOI:** 10.3389/fneur.2018.00136

**Published:** 2018-03-12

**Authors:** Leonidas Trakolis, Florian H. Ebner, Kathrin Machetanz, Joey Sandritter, Marcos Tatagiba, Georgios Naros

**Affiliations:** ^1^Department of Neurosurgery, Eberhardt Karls University, Tuebingen, Germany

**Keywords:** vestibular schwannoma, tinnitus, predictors, hearing impairment, maladaptive neuroplasticity, logistic regression

## Abstract

**Objective:**

Tinnitus is one of the most common symptoms before and/or after the surgical removal of a vestibular schwannoma (VS) affecting almost half of the patients. Although there is increasing evidence for the association of hearing impairment and VS-associated tinnitus, the effect of hearing deterioration due to surgery and its relation to the postoperative tinnitus (postTN) is poorly investigated. This knowledge, however, might (i) enlighten the pathophysiology of VS-associated tinnitus (i.e., peripheral or central origin) and (ii) improve preoperative patient counseling. The aim of this study was to understand the predisposition factors for a postTN in relation to hearing outcome after surgery.

**Methods:**

This retrospective study analyzed the presence of tinnitus in 208 patients with unilateral VS before and after surgical removal. A binomial logistic regression was performed to ascertain the effect of pre- and postoperative hearing as well as age, gender, tumor side, and size, and intraoperative cochlear nerve resection (CNR) on the likelihood of postoperative VS-associated tinnitus.

**Results:**

Preoperative tinnitus was the strongest predictor of postTN. In addition, deterioration of functional hearing was increasing, while functional deafferentation (i.e., postoperative hearing loss) of non-functional hearing was reducing the risk of postTN. At the same time, patients with no preoperative tinnitus but complete hearing loss had the lowest risk to suffer from postTN. Patient age, gender, tumor side, and size as well as CNR played a subordinate role.

**Conclusion:**

While the presence of preoperative tinnitus was the strongest predictor of postTN, there is a distinct relationship between hearing outcome and postTN depending on the preoperative situation. Functional or anatomical deafferentation due to surgical tumor removal does not prevent postTN *per se*.

## Introduction

Tinnitus occurs in 63–75% of patients with unilateral vestibular schwannoma (VS) (1, 2) and in 10% of these patients it is the presenting symptom (3). The pathophysiology of VS-associated tinnitus remains unclear. While some authors suggest a peripheral source, others support a central origin (1, 3). It is hypothesized that the tinnitus initially evolves from an irritation of the cochlear nerve by the tumor (4–6) due to (i) ephaptic coupling of cochlear nerve fibers by compression (7), (ii) cochlear dysfunction by ischemia and biochemical degradation (8), or (iii) efferent system dysfunction following compression of the efferent fibers in the inferior vestibular nerve (9). In the chronic phase, the current hypothesis suggests a maladaptive neuroplasticity on a cochlear, brain stem, and/or cortical level as a consequence of these non-functional signals. The neuroplastic changes are supposed to cause a neuronal hyperexcitability for the residual auditory input resulting in the subjective misperception (10–13). In line with this pathophysiological concept, an association between hearing impairment, i.e., the clinical correlate of damage to the cochlear nerve, and the occurrence of tinnitus in VS patients is expected. However, it was not until recently, that hearing impairment has been shown to predict preoperative VS-associated tinnitus (2). In contrast, complete hearing loss seems to prevent preoperative tinnitus (2, 14, 15). For the postoperative situation, i.e., after removing the irritating agent, preservation of functional hearing is supposed to alleviate the tinnitus. Even more, some authors suggest cochlear nerve resection (CNR) for tinnitus elimination (14, 16).

However, the association between postoperative hearing outcome and tinnitus remains elusive (14, 17–19). While some studies indicate that hearing preservation surgery might prevent a new-onset or improve preoperative tinnitus (17), other studies could not confirm this finding (18). In contrast, an increased incidence of postoperative tinnitus (postTN) in patients with preserved hearing than in patients with postoperative hearing loss has been described (19). Furthermore, the beneficial effect of CNR has not been proven yet (14, 16, 18). In our opinion, this controversy is explained by the suggested pathophysiology of VS-associated tinnitus. As long as there is a peripheral origin of the tinnitus, tumor removal with preservation of cochlear nerve function could improve the tinnitus by eliminating the irritating agent. At the same time, functional (i.e., postoperative ipsilateral hearing loss) or anatomical (i.e., CNR) deafferentation could also eliminate tinnitus by stopping transmission of non-functional signals to the following hearing pathway. In contrast, patients with no preoperative tinnitus whose hearing deteriorates after surgery might have a higher risk to develop tinnitus due to surgical damage of the cochlear nerve. After central maladaptive neuroplasticity took place, however, postoperative hearing deterioration or deafferentation are not expected to influence the tinnitus at all.

We hypothesize that the best predictor of postTN is neither CNR nor hearing outcome itself but the surgery-associated *evolution* of hearing and its relation to the presence of preoperative tinnitus. The aim of the study is to describe the relationship between pre- and postoperative hearing impairment and the occurrence of postTN in VS surgery under the prism of maladaptive neuroplasticity.

## Materials and Methods

### Patients

All patients enrolled in this retrospective cross section study underwent a neurosurgical removal of a unilateral sporadic VS in the Neurosurgical Department of the University of Tuebingen between January 2013 and January 2015. All patients underwent a surgical removal of the tumor *via* a retrosigmoidal-transmeatal approach. Continuous neuromonitoring of the brainstem auditory-evoked potentials (BAEP) was applied in all patients with preserved preoperative BAEP (20, 21). Notably, there was an attempt for anatomical preservation of the cochlear nerve in all patients. After excluding patients with neurofibromatosis II, bilateral VS, relapse or post-radiation surgery, known contralateral hearing loss (Gardner and Robertson grading >2) and incomplete data, we could enroll 208 patients in this retrospective data (47.9 ± 13.1 years, 115 females). Preoperatively, all patients received a clinical evaluation of VS-associated symptoms, a hearing evaluation by an ear–nose–throat specialist [pure tone audiogram and speech discrimination (SDS)] and a magnetic resonance (MR) imaging of the brain. This study was approved by the ethics committee of the Eberhardt Karls University Tuebingen (registration no. 513/2017B02).

### Clinical Evaluation

All patients underwent a thorough clinical evaluation of VS-associated symptoms (i.e., hearing impairment, tinnitus, dizziness, balance problems, facial palsy, facial dysesthesia, swallowing difficulties, headache, nausea, vomiting) by a semi-structured interview by experienced neurosurgeons. Finally, the presence of ipsilateral tinnitus symptoms was dichotomized for statistical analysis (0: no tinnitus, TN−; 1: tinnitus present, TN+). The presence of tinnitus was evaluated preoperatively (preTN) and 3 months postoperatively (postTN).

### Grading of the Hearing Loss

Hearing impairment was classified according to the Gardner and Robertson (GR) scale (22) based on the results of the pure tone audiometry (PTA) and SDS resulting in five classes: GR 1 (good, PTA 0–30 dB, and SDS 70–100%), GR 2 (serviceable, PTA 31–50 dB, and SDS 50–69%), GR 3 (non-serviceable, PTA 51–90 dB, and SDS 5–49%), GR 4 (poor, PTA 51–90 dB, and SDS 1–4%), GR 5 (deaf, PTA 0 dB, and SDS 0%). According to previous publication, GR classification was modified (GR_m_) and hearing impairment was reclassified in (i) GR_m_1: functional hearing (GR1 and GR2), (ii) GR_m_2: non-functional hearing (GR3 and GR4), and (iii) GR_m_3: no hearing (GR5) (14, 18). Hearing grading was performed on PTA and SDS preoperatively (preGR_m_) and postoperatively (postGR_m_). In addition, patients were classified according to their hearing outcome (ΔGR_m_), (i) unchanged hearing preGR_m_1 → postGR_m_1 or preGR_m_2 → postGR_m_2 (ΔGR_m_0), (ii) deterioration of preoperative functional hearing preGR_m_1 → postGR_m_2/3 (ΔGR_m_1), (iii) deterioration of preoperative non-functional hearing preGR_m_2 → postGR_m_3 (ΔGR_m_2), and (iv) preoperative complete hearing loss (ΔGR_m_3).

### Tumor Size Classification

In all patients, a preoperative magnetic resonance image of brain with gadolinium contrast was available and the tumor extent was graded according to Hannover classification (23). VS were classified into four classes: T1 (purely intrameatal), T2 (intra- and extrameatal), T3 (filling the cerebellopontine cistern), T4 (compressing the brain stem). As T1 tumors are often treated non-surgically and underrepresented in neurosurgical cohorts, T1 and T2 tumors are pooled for statistical analysis (T1/2).

### Statistics

All statistical tests were performed using SPSS (IBM SPSS Statistics for Windows, Version 22.0. Armonk, NY, USA: IBM Corp.). Group differences in distribution of clinical attributes such as gender, age, tumor side, tumor size, and preoperative and postoperative hearing impairment were evaluated by Student’s *t*-test or Chi-square test. Binary logistic regression analysis was used to determine the predictive value of gender, age, tumor side, tumor size, the presence of preoperative tinnitus (preTN) and preoperative (preGR_m_) and postoperative (postGR_m_) hearing impairment as well as the surgery associated change of hearing (ΔGR_m_) for the occurrence of postTN using a backward step-wise method. The backwards method removes explanatory variables from the first model, which includes all the specified variables based on the likelihood ratio criterion, which is considered the criterion least prone to error (24). Predictive values of the included variable are provided by their odds ratios (OR) together with the 95% confidence interval (95% CI). Data are shown as mean ± SD. Statistical significance was considered with *p* < 0.05 for each statistical test.

## Results

### Cohort Characteristics

This retrospective study enrolled 208 patients with unilateral VS (47.9 ± 13.1 years, 115 female). 32.2% (67/208) presented a T1/2, 40.4% (84/208) a T3 and 27.4% (57/208) a T4 tumor according to the Hannover classification. Preoperatively, 63% (131/208) of the patients had functional hearing (preGR_m_1), while 19.7% (41/208) presented non-functional hearing (preGR_m_2) and 17.3% (36/208) were deaf (preGR_m_3). 58.7% (122/208) complained about a preoperative tinnitus (preTN+). All patients underwent a surgical removal of the tumor *via* the retrosigmoidal-transmeatal approach supported by continuous neuromonitoring of the BAEP aiming anatomical preservation of the cochlear nerve. However, in 7.2% (15/208), CNR was noted. Postoperatively, functional hearing (postGR_m_1) was preserved in 48.1% (63/131) of patients.

### Clinical Differences in VS Patients With and Without postTN

The clinical characteristics of postTN− and postTN+ patients are summarized in Table 1. Of the 208 patients, 49.0% (102/208) were postTN+. postTN+ patients were significant younger than the postTN- patients. There were no significant differences in gender, tumor side and size, CNR.

**Table 1:** Differences in vestibular schwannoma patients with (postTN+) and without (postTN−) postoperative tinnitus.

		postTN−	postTN+	
		106/208	102/208	
		51.0%	49.0%		

Age		49.8 ± 14.5	46.0 ± 11.2	*t*(206) = 2.11
				*p* = 0.036

Gender	m	46	47	*X*(1) = 0.151
		43.4%	46.1%	*p* = 0.697
	f	60	55	
		56.6%	53.9%	

Side	L	45	47	*X*(1) = 0.277
		42.5%	46.1%	*p* = 0.599
	R	61	55	
		57.5%	53.9%	

Size	T1/2	37	30	*X*(2) = 1.857
		34.9%	29.4%	*p* = 0.395
	T3	38	46	
		35.8%	45.1%	
	T4	31	26	
		29.2%	25.5%	

preTN	preTN−	63	23	*X*(1) = 29.161
		59.4%	22.5%	*p* < 0.001
	preTN+	43	79	
		40.6%	77.5%	

CNR	No	96	97	*X*(1) = 1.596
		90.6%	95.1%	*p* = 0.207
	Yes	10	5	
		9.4%	4.9%	

preGR_m_	PreGR_m_1	55	76	*X*(2) = 15.016
		51.9%	74.5%	*p* = 0.001
	PreGR_m_2	23	18	
		21.7%	17.6%	
	PreGR_m_3	28	8	
		26.4%	7.8%	

postGR_m_	PostGR_m_1	27	36	*X*(2) = 4.743
		25.5%	35.3%	*p* = 0.093
	postGR_m_2	26	30	
		24.5%	29.4%	
	postGR_m_3	53	36	
		50.0%	35.3%	

ΔGR_m_	ΔGRm0	35	46	*X*(3) = 16.782
		33.0%	45.1%	*p* = 0.001
	ΔGR_m_1	28	40	
		26.4%	39.2%	
	ΔGRm2	15	8	
		14.2%	7.8%	
	ΔGRm3	28	8	
		26.4%	7.8%	

### Relationship Between Pre- and postTN in VS Patients

81.4% (83/102) of postTN+ patients were suffering from tinnitus preoperatively (preTN+) but only 40.6% (43/106) of postTN− patients were preTN+. In detail, in 67.5% (85/126) of preTN+ patients, the tinnitus sustained after surgery, while tinnitus disappeared in only 32.5% (41/126) of preTN+ patients. In contrast, 79.3% (65/82) of preTN− patients had no postTN, while 20.7% (17/82) developed a new-onset tinnitus after surgery.

### Relationship Between Hearing Outcome and postTN

Patients with preoperative hearing loss (preGR_m_3) were less likely than patients with residual preoperative hearing [*X*(2) = 15.016; *p* = 0.001] to suffer from postTN. 26.4% (28/106) of postTN− patients had preGR_m_3; however, only 7.8% (8/102) of postTN+ patients had a preoperative hearing loss (preGR_m_3). While there were no significant differences in postoperative hearing level (postGR_m_), there was a significant difference in change of hearing level (ΔGR_m_) due to the surgery. Most postTN+ patients showed unchanged (ΔGR_m_0) or a deterioration of preoperative functional hearing (ΔGR_m_1), while postTN− showed a high rate of postoperative hearing loss (ΔGR_m_2 and ΔGR_m_3; Table 1).

**Figure 1:**
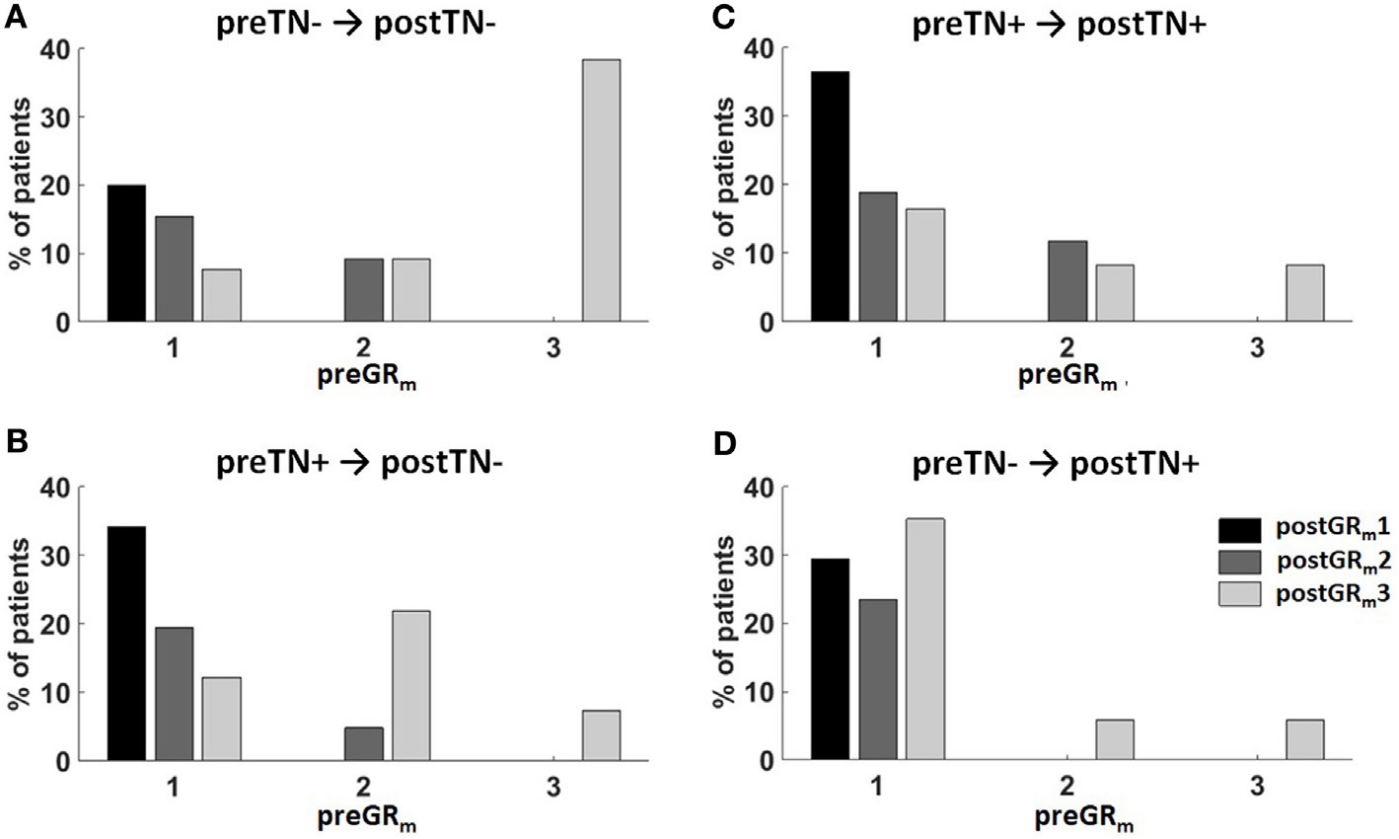
Summary of the surgical hearing and tinnitus outcome. Patients were classified according to the change of their pre- and postoperative tinnitus (postTN) in four groups: **(A)** preTN− → postTN−, **(B)** preTN+ → postTN−, **(C)** preTN+ → postTN+, and **(D)** preTN− → postTN+. The *x*-axis shows the distribution of the preoperative hearing (preGR_m_1: functional hearing; preGR_m_2: non-functional hearing; preGR_m_3: ipsilateral hearing loss) within group. Color coding represents the hearing outcome (black: postGR_m_1; dark gray: postGR_m_2; light gray: postGR_m_3).

The relationship between the hearing and tinnitus outcome after VS surgery is visualized in Figure 1. Notably, preGR_m_3 patients without preoperative tinnitus had a high chance of postTN absence (Figure 1A). In addition, postoperative hearing loss after preoperative non-functional hearing was associated with disappearance of preoperative tinnitus (Figure 1B). In contrast, in patients with preoperative functional hearing and postoperative unchanged or deteriorated hearing tinnitus persisted postoperatively (Figure 1C) or even a new-onset tinnitus (Figure 1D) occurred.

### Prediction of postTN

In order to ascertain the effects of age, gender, tumor side, and size, the presence of preoperative tinnitus (preTN), CNR, pre- (preGR_m_), and postoperative hearing (postGR_m_) as well as the hearing change (ΔGR_m_) on the likelihood of postoperative VS-associated tinnitus, a binomial logistic regression was performed using a backward step-wise method. After seven iterations, the logistic regression model was statistically significant [χ^2^_(3)_ = 41.08, *p* < 0.001]. Of the predictor variables, only preTN and ΔGR_m_ were included in the model as significant predictors of postTN (Table 2). Patients with preoperative tinnitus had a significantly higher risk to suffer from postTN [odds ratio (OR) 4.63 (2.44–8.77); *p* < 0.001]. In contrast, preoperative hearing loss (ΔGR_m_3) and postoperative hearing loss in patients with preoperative non-functional hearing (ΔGR_m_2) are reducing the risk of postTN [OR 0.35 (0.14–0.93); *p* = 0.035 and OR 0.32 (0.12–0.89); *p* = 0.029]. In turn, this means that patients with unchanged hearing or deterioration of initial functional hearing have an increased risk of postTN.

**Table 2:** Logistic regression predicting postoperative tinnitus.

	*B*	SE	Wald	Df	*p*	Odds ratio (OR)	95% CI for OR	
							Lower	Upper
preTN	1.532	0.326	22.035	1	0.000	4.626	2.440	8.769
ΔGR_m_			10.447	3	0.015			
ΔGR_m_1	0.133	0.356	0.139	1	0.710	1.142	0.568	2.296
ΔGR_m_2	−1.132	0.518	4.783	1	0.029	0.322	0.117	0.889
ΔGR_m_3	−1.039	0.492	4.465	1	0.035	0.354	0.135	0.928
Constant	−0.712	0.319	4.990	1	0.025	0.490		

*ΔGR_m_ is compared to ΔGR_m_0, preTN is compared to preTN−*.

## Discussion

The aim of this study is to describe the relationship between pre- and postoperative hearing impairment and the occurrence of postTN in VS surgery. While the presence of preoperative tinnitus was the strongest predictor, our data show that patients with preservation or deterioration of preoperative functional hearing have a high risk of postTN. In contrast, functional deafferentation (i.e., postoperative ipsilateral hearing loss) of preoperative non-functional hearing is reducing the risk of postTN, significantly. At the same time, patients with no preoperative tinnitus but complete hearing loss have the lowest risk to suffer from postTN. Patient age, gender, tumor side, and size as well as CNR play a subordinate role in predicting postTN.

### Effect of Tumor Removal on Tinnitus

In the present study, 67.5% of patients with preoperative tinnitus were still suffering after the surgery, while in 32.5%, tinnitus disappeared postoperatively. In contrast, 79.3% of patients with no preoperative tinnitus, while 20.7% of patients developed a new-onset tinnitus. There are several studies evaluating the tinnitus occurrence after VS surgery showing a high variability in tinnitus outcome. This could probably be attributed to difference in surgical procedures and approaches (18). However, our results are in good accordance to studies with a similar surgical procedure (i.e., retrosigmoidal-transmeatal approach). Kameda et al. reported a symptom improvement/stability in 64.9% and a disappearance in 25.2% of the patients with preoperative tinnitus. In contrast to our results, they described that 91.5% remained symptom-free after surgery while only 8.5% of the patients developed a new-onset tinnitus (18). Considering the comparable preservation rates of useful hearing in both studies (51.9 vs 48.1% in the present study), it remains unclear, whether the lower rate of new-onset tinnitus is attributed to (i) the CNR (53.7 vs 7.2% in the present study), (ii) to differences in preoperative hearing level (preoperative functional hearing in 42.6 vs 63.0% in the present study), (iii) to the preoperative tinnitus of the patients (70.7 vs 58.7% in the present study), or (iv) to the smaller tumor size (48.5% <2 cm vs 32.2% T1/2 in the present study) (18). For comparison, Chovanec et al. report a disappearance of preoperative tinnitus in 66% but a new-onset tinnitus in 14% of the patients while preserving preoperative hearing level in 19.1% (14).

### Relationship Between Postoperative Hearing Outcome and Tinnitus

It was not until recently that the correlation between non-functional hearing, i.e., the clinical correlate of incomplete cochlear nerve impairment, and the occurrence of preoperative tinnitus in VS was shown (2). In contrast, complete hearing loss prevented tinnitus (2, 14, 15). There is still a controversy concerning the hearing outcome and tinnitus after VS surgery (14, 17–19). Our data show that the occurrence of postTN is predicted best by the *evolution* of hearing after surgery than by the pre- or postoperative hearing level. Patients with hearing preservation or deterioration of preoperative functional hearing have the highest risk, while functional deafferentation of preoperative non-functional hearing and preoperative hearing loss is reducing the risk of postTN. Among the studies that performed a similar surgical strategy, none have shown a significant association between the postoperative hearing outcome and tinnitus (14, 18). In contrast to our study, Chovanec et al. have shown a significant higher prevalence of postTN in cases with anatomically preserved cochlear nerve but postoperatively deafened ear. Additionally, there was a significant higher prevalence of tinnitus elimination in cases of CNR (14). Although not reaching statistical significance, the authors describe several observations that correspond to our findings. More specifically, postTN was more prevalent in patients with preoperative hearing than in preoperatively ipsilateraly deafened patients. Notably, there was no new-onset tinnitus in patients with preoperative hearing loss. New-onset tinnitus had the highest prevalence in the group of preserved non-functional hearing. The incidence of postTN was lowest in patients with postoperative functional hearing or in patients with preoperative hearing loss (14).

To our opinion, these findings support the current pathophysiological concept of tinnitus. Tinnitus initiation is supposed to evolve from an irritation of the cochlear nerve by the tumor and the consecutive non-functional afference to central hearing system (2, 6). In line, there is recent evidence that non-functional hearing predicts the occurrence (2) while hearing loss prevents VS-associated tinnitus (2, 14, 15). In the non-chronic phase, removing the non-functional input by functional or anatomical deafferentation will improve preoperative tinnitus. This hypothesis is supported by our data showing that postoperative hearing loss after preoperative non-functional hearing is reducing the risk of postTN. In contrast, deterioration of a preoperative functional hearing by damage to the cochlear nerve has a high risk to trigger new-onset tinnitus after surgery. In cases of centralization of the tinnitus, however, non-functional input to the central hearing system is supposed to cause maladaptive neuroplasticity resulting in neuronal hyperexcitability and auditory misperceptions (10–13). Furthermore, the strongest predictor of postTN is the presence of a preoperative tinnitus independent of the hearing outcome. Additionally, patients suffering of preoperative tinnitus and hearing loss do have a high prevalence of postTN. Finally, patients with no preoperative tinnitus but complete hearing loss have the lowest risk to suffer from postTN. We hypothesize that in case functional deafferentation occurs in a short period of time (i.e., acute hearing loss), there is no time for neuroplasticity to take place.

### Impact of CNR on VS-Associated Tinnitus

Based on the idea to remove non-functional afference to the central hearing system, some authors suggest CNR for tinnitus alleviation (14, 16). Although the effectivity of this procedure has not been proven yet (14, 18), our data support the hypothesis that anatomical deafferentation of the cochlear nerve might reduce the risk of postTN in patients where no functional postoperative hearing is expected (14). However, this is not the standard of care. There are several good reasons to preserve anatomical cochlear nerve integrity, even in patients with clearly reduced ipsilateral hearing or hearing loss. (i) It is difficult to predict definite postoperative hearing outcome despite the application of intraoperative neuromonitoring of BAEP (14, 25). So far, there is no study showing any predictive value of intraoperative BAEP for postTN. (ii) In case maladaptive neuroplasticity took place, CNR will not improve tinnitus (18). (iii) CNR hampers a later implantation of cochlear implant, which improves hearing (26) and has been shown to reduce tinnitus itself (27) probably by reinstalling functional signal transmission.

### Limitations of the Study

A major limitation of the study is the dichotomization of the patients’ tinnitus complaints. Due to the retrospective design of the study, there is no systematic data on tinnitus severity, frequency, and grade of handicap to the patients suffering as well as the temporal relation between tinnitus onset and hearing impairment or loss. This information would enable conclusions about the modulation of tinnitus severity by the surgical intervention and the time for maladaptive neuroplasticity to take place. Here, we see definite need for further prospective studies.

## Conclusion

Our study is one of the few studies evaluating tinnitus after surgical VS removal *via* the retrosigmoidal-transmeatal approach showing a significant correlation between hearing outcome and postTN. Functional deafferentation of preoperative non-functional hearing and preoperative ipsilateral hearing loss are reducing the risk of postTN, while deterioration of preoperative functional hearing and preoperative tinnitus predict postTN persistence or new-onset tinnitus. This information might help the surgeons not only during the preoperative counseling and the consent of the patient but also to develop a better surgical strategy. The possibility and probability of postTN, together with the possible reduction in quality of life must be thoroughly discussed with the patient before surgery.

## Ethics Statement

This study was approved by the ethics committee of the Eberhardt Karls University Tuebingen (registration no. 513/2017B02).

## Author Contributions

LT contributed to the analysis and interpretation of data, statistical analysis, and writing of the first draft. JS, KM, FE, and MT contributed to the data acquisition, interpretation of data, and the review and critique of the final manuscript. GN was responsible for the conception and design, data acquisition, analysis and interpretation, statistical analysis, as well as the review and critique of the manuscript.

## Conflict of Interest Statement

The authors declare that the research was conducted in the absence of any commercial or financial relationships that could be construed as a potential conflict of interest.

## Abbreviations

CI, confidence interval; ENT, ear–nose–throat; GR, Gardner and Robertson scale; LR, likelihood ratio; MRI, magnetic resonance image; NF, neurofibromatosis; OR, odds ratio; PTA, pure tone audiometry; SDS, speech discrimination score; VS, vestibular schwannoma.
